# Associations Between Body Composition and Intratumoural Immune Cells in Patients With Uterine Cervical Cancer

**DOI:** 10.1002/jcsm.70241

**Published:** 2026-03-10

**Authors:** Alexey Surov, Anne‐Katrin Höhn, Mattes Hinnerichs, Hans‐Jonas Meyer, Jan Borggrefe

**Affiliations:** ^1^ Institute of Radiology, Neuroradiology and Nuclear Medicine, Johannes‐Wesling‐Klinikum Minden, Ruhr‐University Bochum Bochum Germany; ^2^ Department of Pathology University Hospital of Leipzig Leipzig Germany; ^3^ Department of Radiology University Hospital of Leipzig Leipzig Germany; ^4^ Department of Radiology and Nuclear Medicine University Hospital of Magdeburg Magdeburg Germany

**Keywords:** body composition, cervical cancer, tumoural immune cells

## Abstract

**Background:**

In uterine cervical cancer (UCC), reduced muscle mass and radiodensity have been linked to unfavourable clinical outcomes. Our aim was to elucidate the associations between body composition (BC) and intratumoural immune cells in patients with UCC.

**Methods:**

In this retrospective study, BC was analysed in 61 patients with UCC using staging computed tomography. The parameters of BC were skeletal muscle area (SMA), skeletal muscle radiodensity, visceral adipose tissue (VAT), subcutaneous adipose tissue (SAT) and intramuscular adipose tissue (IMAT). The radiodensities of VAT, SAT and IMAT were also estimated. Tumour specimens underwent histopathological analysis to quantify stromal and intratumoural CD45‐positive cells. Associations between body composition parameters and tumour immune cell infiltration were analysed using ANOVA.

**Results:**

Patients with low muscle radiodensity (myosteatosis) had a lower proportion of stromal CD45‐positive cells than patients with normal muscle radiodensity (21.6% ± 21.90% vs. 34.85% ± 25.55%; *p* = 0.04). High levels of SAT were associated with lower scores for tumour‐infiltrating CD45 cells (*p* = 0.03). High IMAT radiodensity was associated with lower stromal CD45 scores (*p* = 0.02).

**Conclusions:**

Myosteatosis, high SAT and increased IMAT radiodensity were associated with reduced stromal and intratumoural immune cell infiltration in patients with UCC. These body composition parameters may serve as prognostic markers and should be explored in risk stratification.

**Abbreviations**
CT: computed tomography
IMAT: intramuscular adipose tissue
LSMM: low skeletal muscle mass
RFS: recurrence‐free survival
SAT: subcutaneous adipose tissue
SMA: skeletal muscle area
SMD: skeletal muscle (radio)density
SIC: stromal immune cells
TIC: tumoural immune cells
TIL: tumour‐infiltrating lymphocytes
UCC: uterine cervical cancer
VAT: visceral adipose tissue
VSR: visceral to subcutaneous ratio of adipose tissue

## 1 Introduction

Uterine cervical cancer (UCC) is one of the most common malignancies in women and ranks as the fourth leading cause of female cancer‐related deaths worldwide [1]. Accurate prediction of clinical outcomes is essential in UCC, as it helps to ensure that patients receive the best possible care and treatment. Cross‐sectional imaging, such as computed tomography (CT), provides data for tumour staging and valuable insight into the patient's general health [2, 3]. In particular, CT can be used for the analysis of body composition, i.e., proportion, distribution and quality of muscle and adipose tissues in the body [3]. Several studies have demonstrated that body composition parameters can predict relevant outcomes in patients with UCC [4, 5, 6]. For example, low skeletal muscle mass (LSMM) prior to treatment is strongly and independently associated with poorer overall survival (OS; hazard ratio [HR] = 3.09; 95% confidence interval [CI], 2.07–4.61; *p* < 0.00001 and progression‐free survival, HR = 1.55; 95% CI, 1.06–2.28; *p* = 0.03) [5]. Furthermore, skeletal muscle loss during chemotherapy is independently linked to worse disease‐free survival (HR = 2.96; *p* = 0.006) [4]. Also skeletal muscle loss during concurrent chemoradiotherapy is strongly and independently associated with poorer OS (HR = 5.18; 95% CI, 3.54–7.56; *p* < 0.00001) [5, 7]. Elevated levels of visceral adipose tissue (VAT) have been identified as an independent negative prognostic factor for disease‐specific survival in UCC (HR = 1.04; 95% CI, 1.00–1.08; *p* = 0.03) [8].

Although these associations are well documented, the underlying pathomechanisms remain poorly understood. Emerging evidence suggests that body composition may influence immune cell activity in the tumour microenvironment and could thus represent a relevant pathophysiological mechanism of the host metabolism to influence tumour immunogenicity and growth [9]. However, empirical evidence supporting this hypothesis is still limited.

Therefore, the aim of this study was to investigate possible associations between CT‐based body composition parameters and tumour‐infiltrating immune cells in patients with cervical cancer (UCC).

## 2 Methods

### 2.1 Patient Acquisition

This retrospective study was performed after approval of the local ethics committee and conducted in accordance with the ethical standards of the institutional and/or national research committee and with the Declaration of Helsinki (ethical code: 012/13‐28 012 013).

**FIGURE 1:**
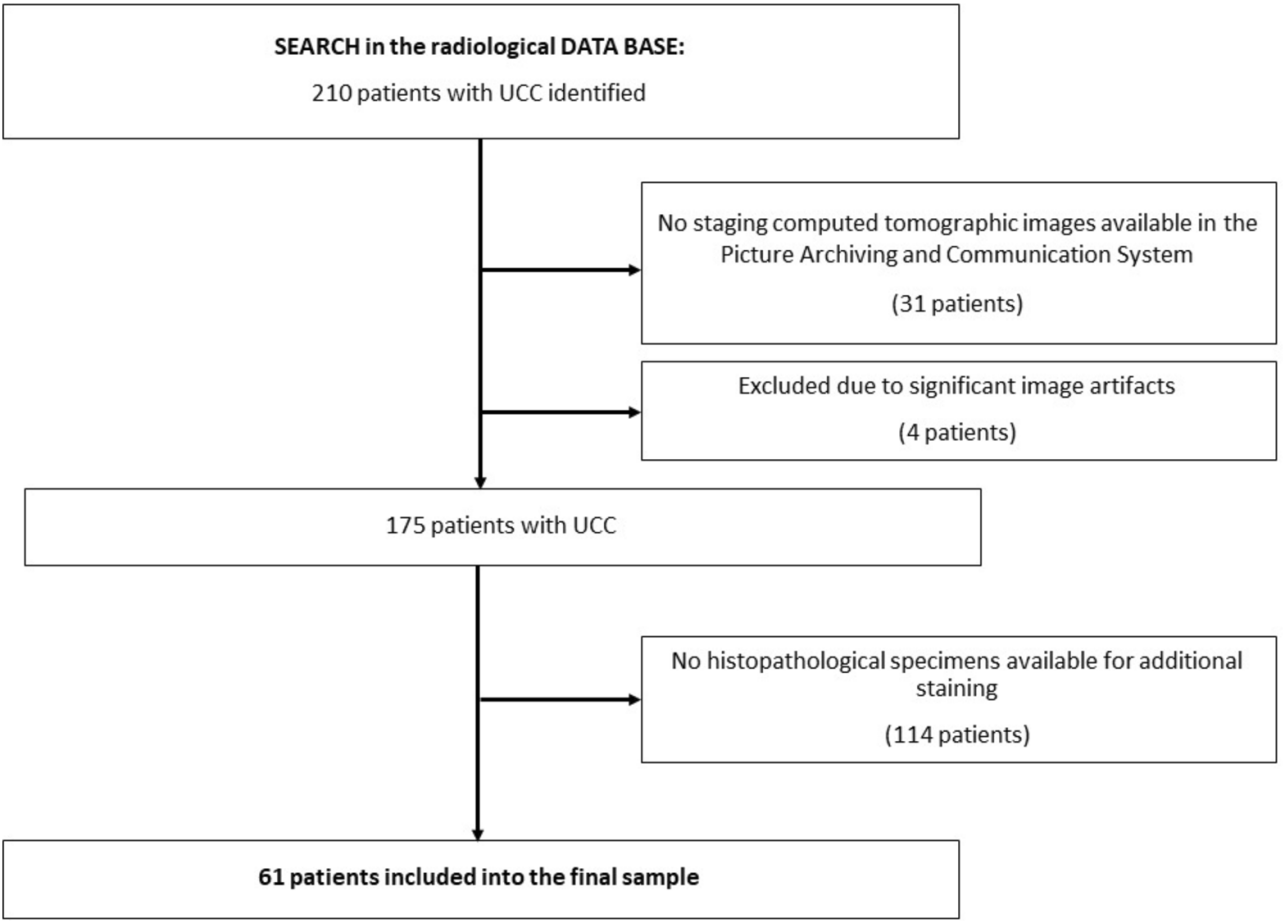
Flow chart of data acquisition.

All consecutive patients diagnosed with UCC at our tertiary referral hospital in the time period from 2014 to 2017 were retrospectively assessed. For this purpose, a computerized search for cases with UCC was performed in the radiological database (Figure 1). A total of 210 patients were identified.

Inclusion criteria for the study were:

- Availability of contrast‐enhanced staging baseline CT scans at the time of initial diagnosis
- Histopathological confirmation of squamous cell UCC
- Availability of unstained histopathological tissue specimens

Exclusion criteria were:

- Incomplete imaging or histopathological data
- Nonsquamous histological tumour types
- Relevant imaging artefacts that affected the analysis

### 2.2 Imaging Technique and Analysis of Body Composition

**FIGURE 2:**
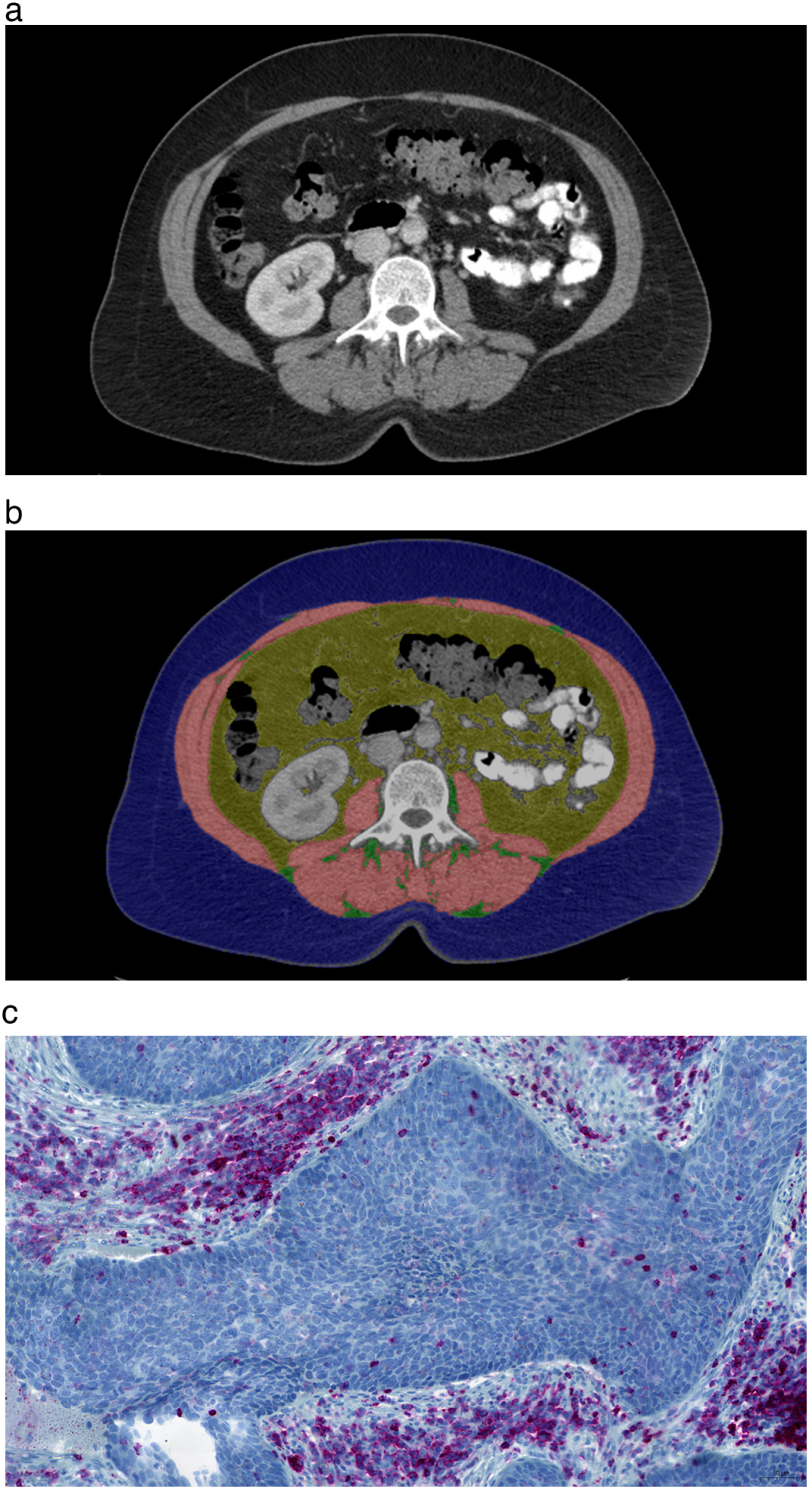
Imaging and histopathological findings in a 55‐year‐old patient with UCC. (a) Staging computed tomographic scan. (b) Segmented area of body composition: skeletal musculature (red), visceral adipose tissue (yellow), SAT (blue) and IMAT (green). (c) Histopathological features (CD45 stained image): 40% of the stromal cells are CD45‐positive cells (SIC score 4) and 1% of the tumoural cells are CD45‐positive (TIC score 1).

All CT scans were obtained using a clinical 256‐slice CT scanner (iCT, Philips, Amsterdam, the Netherlands). Imaging parameters were 120 kVp, automatic tube current modulation (150–300 mAs) and 1 mm slice thickness. CT images were acquired in portal venous phase 40 s after intravenous injection of contrast media in all cases (Figure 2a).

Body composition parameters were semiautomatically measured with the freely available ImageJ software (version 1.53, National Institutes of Health, USA) by one board‐certified radiologist (M.H., with 5 years of general radiological experience), blinded to the histopathological findings in accordance with the previous descriptions [10]. All measurements were taken from a single axial CT‐slice at the level of the middle of the third lumbar vertebral body (Figure 2a) [11]. The skeletal muscle area (SMA) was defined using attenuation threshold levels of −29 to +150 Hounsfield units (HU), and adipose tissue compartments, namely, VAT, subcutaneous adipose tissue (SAT) and intramuscular adipose tissue (IMAT), were quantified separately using threshold levels of −190 to −30 HU (Figure 2b) [11]. The SMA threshold of 92.2 cm^2^ proposed by Derstine et al. [12] was used for the definition of LSMM or sarcopenia. An area measuring more than 100 cm^2^ was defined as high VAT and high SAT. The VAT/SAT ratio was calculated, and values greater than 1.1 were classified as elevated. A high IMAT level was defined as a value that exceeded the median (9.2 cm^2^). Low skeletal muscle radiodensity (SMD), also known as myosteatosis, was considered if the level was below 34.3 HU [12]. The median values were used as thresholds for adipose tissue radiodensity. They were as follows: SAT: −98 HU, IMAT: −62 HU, and VAT: −79 HU.

### 2.3 Histopathological Analysis

For the study, bioptic histopathological samples were analysed. All were samples obtained prior to therapy initiation. Formalin‐fixed, paraffin‐embedded tissue serial sections (2 μm) were dewaxed in xylol and rehydrated by descending concentrations of ethanol. For antigen detection, the automated immunohistochemistry slide staining system VENTANA BenchMark ULTRA (Roche Diagnostics GmbH), the VENTANA iVIEW DAB Detection Kit (Roche Diagnostics GmbH) following antigen retrieval was with CC1 mild buffer. The slides were then incubated with specific primary antibodies recognizing CD45 (polyclonal mouse antibody, clone 2B11 + PD7/26; DAKO/Agilent #M0701). Histopathological slides were digitalized and saved as uncompressed tagged image file format (TIFF).

Image analysis of histopathological slides was conducted by a single experienced investigator (AKH with 18 years of experience) blinded to clinical and radiological data. In each case, two power fields (0.20 mm^2^ at 40× magnification) were selected for the analysis. Total CD45 cell count was estimated as a mean value of cells in two power fields [13]. In a similar manner, a mean value of stromal (SIC) and tumoural (TIC) CD45‐positive cells was calculated (Figure 2c) [13]. Finally, a score of CD45‐positive cells was calculated for stromal and tumoural cells as follows:

0 points: no positive cells; 1 point: a few sparsely scattered individual positive cells; 2 points: scattered individual positive cells and small cell clusters with approximately 5% of all cells staining positively; 3 points: medium clusters of positive cells with approximately 10% of all cells staining positively; 4 points: abundant staining across the sample with more than 20% of all cells staining positively [14].

### 2.4 Statistical Analysis

Statistical analysis was performed using the SAS Jmp pro 18.0.2 (SAS Institute, Cary, NC, USA). The collected data were evaluated by means of descriptive statistics (absolute and relative frequencies). Comparison of body composition parameters and tumour‐infiltrating lymphocytes (TILs) was performed by Wilcoxon tests. All *p*‐values are interpreted in an exploratory sense.

## 3 Results

The final study cohort included 61 patients with histologically confirmed squamous cell cervical carcinoma (age range, 32–79 years; mean age, 55.4 years). An overview of clinical characteristics of the patient sample is provided in Table 1. A detailed overview of the analysed parameters of body composition and tumour histopathology is summarized in Tables 2 and 3.

**TABLE 1:** Included patients and tumours.

Age, M ± SD, median	50.2 ± 12.6; 51
Tumour stage	*n* (%)
T1	14 (23)
T2	31 (51)
T3	8 (13)
T4	8 (13)
Nodal stage	
N0	22 (36)
N+	39 (64)
M stage	
M0	46 (75)
M+	15 (25)
Tumour grade	
FIGO stage	
IB1	20 (33)
IB2	5 (8)
IIA	2 (3)
IIB	19 (31)
IIIA	5 (8)
IIIB	9 (15)
IVA	1 (2)

**TABLE 2:** Parameters of body composition in the sample.

Parameters	M ± SD; median
SMA, cm^2^	107.4 ± 18.5; 105.6
Skeletal muscle radiodensity, HU	34.6 ± 9.6; 36.3
VAT area, cm^2^	92.9 ± 76.8; 79.4
VAT density, HU	−77.9 ± 14.7; −78.9
SAT area, cm^2^	209.8 ± 136.2; 187.1
SAT radiodensity, HU	−93.2 ± 16.5; −98.2
IMAT radiodensity, HU	12.9 ± 11.6; 9.2
IMAT radiodensity, HU	−60.9 ± 7.2; −61.6
Body composition	Patients, *n* (%)
LSMM	24 (39)
Myosteatosis or low muscle radiodensity	13 (21)
High VAT	24 (39)
High SAT	48 (78.7)
High IMAT	30 (49.2)
High VAT radiodensity	31 (50.8)
High SAT radiodensity	30 (49.2)
High IMAT radiodensity	31 (50.8)

Abbreviations: IMAT, intramuscular adipose tissue; LSMM, low skeletal muscle mass; SAT, subcutaneous adipose tissue; VAT, visceral adipose tissue.

**TABLE 3:** Intratumoural immune cells (CD45).

Parameters	M ± SD; median; range
SIC, *n*	31.6 ± 25.2; 30; 1–80
SIC score	3.3 ± 0.9; 4; 1–4
TIC, *n*	2.8 ± 3.1; 1; 1–10
TIC score	1.4 ± 0.7; 1; 1–3

Abbreviations: SIC, stromal immune cells; TIC, tumoural immune cells.

**FIGURE 3:**
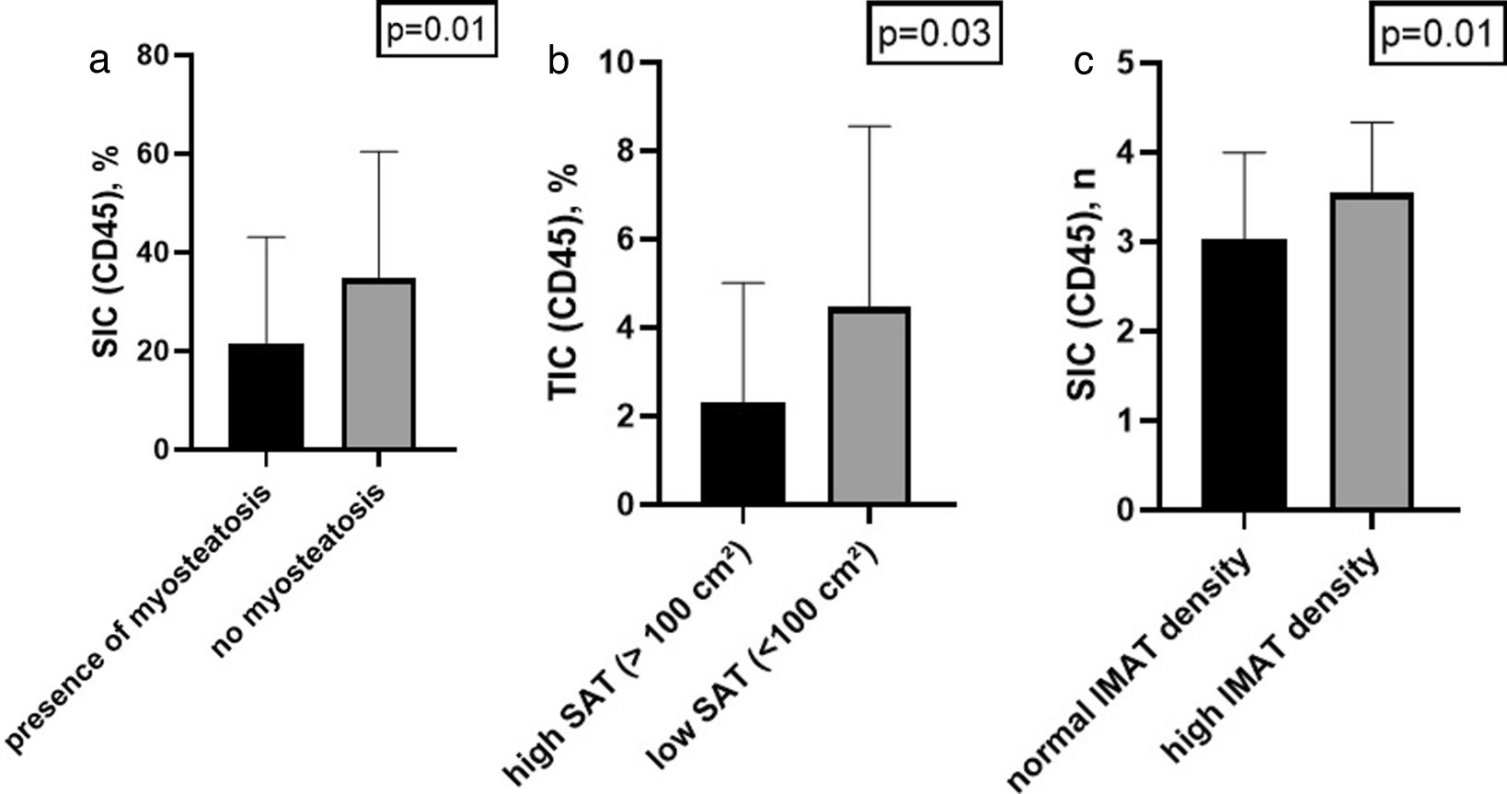
Comparison of intratumoural CD45‐positive cells in patients with different parameters of body composition. (a) SICs in patients with myosteatosis in comparison to patients with normal muscle radiodensity. (b) TICs in patients with high vs. normal SAT. (c) SIC score in patients with high vs. normal IMAT radiodensity.

Associations between CD45‐positive cells and parameters of body composition are shown in Table 3. Patients with myosteatosis (i.e., low skeletal muscle radiodensity) showed a significantly lower proportion of stromal immune cells (SIC) compared to patients with normal muscle density (21.6% ± 21.9% vs. 34.85% ± 25.55%; *p* = 0.04; Figure 3a). Similarly, patients with high SAT exhibited a significantly lower proportion (*p* = 0.03) and score (*p* = 0.04) of tumoural immune cells (TIC) compared to patients with normal SAT (Figure 3b). Furthermore, patients with high IMAT radiodensity had significantly lower SIC scores (*p* < 0.02; Figure 3c).

No significant associations were observed between other body composition parameters (e.g., SMA, VAT or VSR) and tumoural CD45 cell infiltration (Table 4).

**TABLE 4:** Comparison of TIC infiltration across body composition groups.

	Parameters of body composition		
TICs	LSMM	Normal skeletal muscle mass	*p*
SIC, %	20.93 (±19.8)	35.06 (±25.9)	*p* = 0.07
SIC score	2.93 (±1.0)	3.39 (±0.0.8)	*p* = 0.09
TIC, %	2.47 (±3.2)	2.87 (±3.1)	*p* = 0.39
TIC score	1.33 (±0.7)	1.41 (±0.7)	*p* = 0.60
	Myosteatosis	Normal muscle radiodensity	
SIC, %	21.6 (±21.9)	34.85 (±25.5)	*p* = 0.04
SIC score	2.80 (±1.1)	3.43 (±0.8)	*p* = 0.03
TIC, %	1.86 (±0.8)	3.07 (±0.4)	*p* = 0.11
TIC score	1.20 (±0.6)	1.45 (±0.7)	*p* = 0.19
	High VAT	Normal VAT	
SIC, %	31.25 (±25.9)	31.81 (±25.0)	*p* = 0.91
SIC score	3.25 (±0.8)	3.30 (±0.9)	*p* = 0.66
TIC, %	3.46 (±3.4)	2.32 (±2.9)	*p* = 0.07
TIC score	1.54 (±0.8)	1.30 (±0.7)	*p* = 0.13
	High SAT	Normal SAT	
SIC, %	28.69 (±23.9)	42.30 (±27.6)	*p* = 0.11
SIC score	3.21 (±0.9)	3.54 (±0.8)	*p* = 0.24
TIC, %	2.31 (±2.7)	4.46 (±4.1)	*p* = 0.03
TIC score	1.29 (±0.6)	1.77 (±0.9)	*p* = 0.04
	High IMAT	Normal IMAT	
SIC, %	33.45 (±27.0)	29.67 (±23.4)	*p* = 0.77
SIC score	3.3 (±0.8)	3.25 (±1.0)	*p* = 0.81
TIC, %	2.97 (±3.2)	2.58 (±3.1	*p* = 0.44
TIC score	1.43 (±0.7)	1.35 (±0.7)	*p* = 0.57
	High IMAT radiodensity	Normal IMAT radiodensity	
SIC, %	38.10 (±25.3)	25.68 (±23.1)	*p* = 0.06
SIC score	3.03 (±0.9)	3.55 (±0.8)	*p* = 0.02
TIC, %	3.13 (±3.3)	2.38 (±2.9)	*p* = 0.28
TIC score	1.31 (±0.6)	1.46 (±0.7)	*p* = 0.36
	High VAT radiodensity	Low VAT radiodensity	
SIC, %	26.96 (±23.4)	36.06 (±26.1)	*p* = 0.57
SIC score	3.13 (±0.93)	3.42 (±0.89)	*p* = 0.16
TIC, %	2.97 (±3.18)	2.58 (±3.13)	*p* = 0.44
TIC score	1.43 (±0.72)	1.35 (±0.71)	*p* = 0.57
	High SAT radiodensity	Low SAT radiodensity	
SIC, %	35.93 (±25.6)	27.38 (±24.4)	*p* = 0.22
SIC score	3.40 (±0.89)	3.16 (±0.9)	*p* = 0.24
TIC, %	2.76 (±0.6)	2.77 (±0.6)	*p* = 0.99
TIC score	1.40 (±0.7)	1.39 (±0.7)	*p* = 0.94

Abbreviations: IMAT, intramuscular adipose tissue; LSMM, low skeletal muscle mass; SAT, subcutaneous adipose tissue; SIC, stromal immune cells; TIC, tumoural immune cells; VAT, visceral adipose tissue.

## 4 Discussion

To the best of our knowledge, this is the first study to investigate associations between CT‐derived body composition parameters and tumour‐infiltrating immune cells in cervical cancer. Our findings reveal significant relationships between skeletal muscle composition, SAT and density of IMAT with the density of CD45‐positive cells within the tumour microenvironment.

Previous studies have described associations between body composition and clinical outcomes in UCC, including overall and disease‐free survival [5, 6, 7, 8]. Several mechanisms have been proposed to explain these relationships [4, 5, 6, 7, 8]. First, body composition parameters reflect the general nutritional and metabolic status [5, 6, 7]. It is well known that LSMM is associated with malnutrition and protein deficiency [5, 6]. According to the literature, patients with UCC are at increased risk of malnutrition [5, 6, 7]. Also, anticancer treatments frequently cause toxicities to the gastrointestinal tracts and provoke disease‐related cachexia [5, 6, 7]. Second, LSMM, myosteatosis and visceral adiposity are risk factors for insulin resistance and impaired glucose metabolism [15, 16]. Third, LSMM and high adipose tissue are associated with increased oxidative stress [16, 17]. Additionally, IMAT and VAT secrete numerous proinflammatory cytokines and angiogenic factors such as adipocyte‐induced insulin‐like growth factor, interleukin‐6 and tumour necrosis factor‐α, all of which promote systemic inflammation, tumour progression and cachexia [18, 19]. Finally, LSMM, myosteatosis and high IMAT are strongly associated with systemic inflammation and correlate with the level of inflammatory markers like C‐reactive protein and neutrophil–platelet score [20].

However, these mechanisms offer only indirect explanations for the association between body composition parameters and cancer progression. They can explain how body composition influences OS, but not how it influences tumour progression, progression‐free survival, or recurrence‐free survival. Our study adds novel evidence by showing that body composition—specifically myosteatosis, high SAT and increased IMAT radiodensity—is directly associated with a reduced density of tumour‐associated immune cells. This finding is very important as it suggests that altered body composition may influence the local tumour immune milieu, thereby contributing to immune escape and tumour progression. According to the literature, TILs are relevant predictors of clinical outcomes in several tumours [21, 22]. Thus far, in a large meta‐analysis including 6647 cancer patients, a high proportion of TILs expression was associated with longer OS (HR = 0.66; 95% CI, 0.50–0.86; *p* = 0.002) and recurrence‐free survival (HR = 0.61; 95% CI, 0.47–0.79; *p* = 0.0001) [21]. Moreover, high TIL levels in tumours were associated with a low risk of recurrence (HR = 0.43; 95% CI, 0.32–0.57; *p* < 0.0001) [21]. In UCC, TILs also have a significant impact on clinical outcomes [23, 24, 25]. In patients with UCC who are undergoing definitive radiotherapy treatment, high TIL expression is associated with longer progression‐free survival (HR = 0.39; 95% CI, 0.21–0.72; *p* = 0.0027) [26]. Higher levels of TILs are also independently associated with favourable OS (HR = 0.71; 95% CI, 0.57–0.89; *p* = 0.003) [25].

Our study offers a pivotal insight, providing a missing direct link between body composition and clinical outcomes in patients with UCC. The identified phenomenon can be explained by endocrine function of skeletal muscle tissue. Muscle fibres synthesize and secrete cytokines, so called myokines, which influence the antitumoural immunity [27]. For example, the myokine interleukin‐15 is a myokine known to stimulate the proliferation of natural killer (NK) cells and CD8+ T lymphocytes, both key effectors of antitumour immunity [27, 28]. In fact, clinical administration of IL‐15 has been shown to induce the proliferation of CD8 cells and NK cells in both circulation and tumour tissue [28]. Our results support the notion that the skeletal muscle composition, i.e., the presence of myosteatosis and/or high IMAT, is crucial for maintaining local immune competence in cervical cancer.

Interestingly, we also observed a negative association between SAT and the intratumoural immune cell infiltration. This finding has not been previously reported and warrants further investigation. In contrast, VAT, despite its known proinflammatory and protumourigenic properties, was not associated with the number of intratumoural immune cells in our cohort. Similar results were observed recently by Ravensbergen et al. [29] in colorectal cancer, suggesting that VAT may exert systemic rather than local immunogenic effects.

Our data are in line with previous observations in other malignancies. For example, in cholangiocarcinoma, patients with LSMM had a lower number of TILs than patients without LSMM [30]. Similar results were reported in patients with colorectal cancer, hepatocellular carcinoma and pancreatic adenocarcinoma [31, 32, 33].

Several limitations of our study must be acknowledged. First, the retrospective design introduces a potential selection bias. Second, the sample size was relatively small. Third, we relied on CT‐derived surrogate measures of body composition rather than functional metabolic assessments. Finally, we analysed the proportion of CD45‐positive cells to identify the cumulative tumoural immunity. CD45 is known as the leukocyte common antigen [34]. Previous studies about the prognostic role of TILs in oncology investigated different subpopulations of lymphocytes: CD3, CD4, CD8, CD14, CD20, CD45, CD56, CD103, CD203 [21, 22, 34, 35, 36, 37, 38, 39]. Predominantly, CD3, CD4 and CD8 cells were analysed [21, 22, 36]. Clearly, further studies analysing different subsets of TILs are needed. Despite these limitations, our findings offer a novel and plausible link between body composition and tumour immunobiology in cervical cancer. In fact, parameters of body composition are modifiable factors. For instance, exercise and nutritional support may improve muscle composition in cancer patients [40]. Therefore, a quantitative analysis of body composition and development of supportive regimes may be beneficial for patients with UCC. Our results suggest the potential importance of personalized supportive care, including tailored nutrition and exercise interventions. Future research should investigate whether prehabilitation strategies targeting muscle and adipose tissue could enhance tumour immune responses and improve clinical outcomes in UCC.

In conclusion, myosteatosis, elevated SAT and increased IMAT radiodensity are associated with reduced stromal and TIC infiltration and are thus risk factors for patients with UCC. These findings suggest that body composition parameters may serve as immune‐relevant prognostic markers and should be explored in future risk stratification and supportive care strategies for affected patients.

## Funding

The authors have nothing to report.

## Conflicts of Interest

The authors declare no conflicts of interest.

## References

[1] R. L. Siegel , T. B. Kratzer , A. N. Giaquinto , H. Sung , and A. Jemal , “Cancer Statistics, 2025,” CA: A Cancer Journal for Clinicians 75, no. 1 (2025 Jan–Feb): 10–45. 39817679 10.3322/caac.21871 PMC11745215

[2] B. D. Pooler , J. W. Garrett , M. H. Lee , et al., “CT‐Based Body Composition Measures and Systemic Disease: A Population‐Level Analysis Using Artificial Intelligence Tools in Over 100,000 Patients,” American Journal of Roentgenology 224, no. 3 (2025): e2432216. 39772583 10.2214/AJR.24.32216

[3] J. Han , L. Harrison , L. Patzelt , et al., “Imaging Modalities for Diagnosis and Monitoring of Cancer Cachexia,” EJNMMI Research 11, no. 1 (2021): 94. 34557972 10.1186/s13550-021-00834-2 PMC8460705

[4] M. Sánchez , D. Castro‐Eguiluz , J. Luvián‐Morales , et al., “Deterioration of Nutritional Status of Patients With Locally Advanced Cervical Cancer During Treatment With Concomitant Chemoradiotherapy,” Journal of Human Nutrition and Dietetics 32, no. 4 (2019 Aug): 480–491. 30938007 10.1111/jhn.12649

[5] F. Wang , H. Zhen , K. Yu , and P. Liu , “The Prognostic Value of Sarcopenia in Clinical Outcomes in Cervical Cancer: A Systematic Review and Meta‐Analysis,” Journal of Cachexia, Sarcopenia and Muscle 16, no. 1 (2025): e13674. 39797562 10.1002/jcsm.13674 PMC11724193

[6] Y. X. Li , W. W. Xia , and W. Y. Liu , “The Influence Process of Sarcopenia on Female Cancer: A Systematic Review and Meta‐Analysis,” Journal of Obstetrics and Gynaecology Research 47, no. 12 (2021): 4403–4413. 34496449 10.1111/jog.15012

[7] J. Lee , C. L. Chang , J. B. Lin , et al., “Skeletal Muscle Loss Is an Imaging Biomarker of Outcome After Definitive Chemoradiotherapy for Locally Advanced Cervical Cancer,” Clinical Cancer Research 24, no. 20 (2018): 5028–5036. 29959140 10.1158/1078-0432.CCR-18-0788

[8] A. J. Eide , M. K. Halle , N. Lura , et al., “Visceral Fat Percentage for Prediction of Outcome in Uterine Cervical Cancer,” Gynecologic Oncology 176 (2023 Sep): 62–68. 37453220 10.1016/j.ygyno.2023.06.581

[9] S. Doi , S. Yasuda , M. Miyashita , et al., “Prognostic Relevance of Sarcopenia and Tumor‐Infiltrating CD8(+) T Cells in Patients With Hepatocellular Carcinoma,” Annals of Gastroenterological Surgery 9, no. 2 (2024): 359–368. 40046516 10.1002/ags3.12875 PMC11877349

[10] A. Surov , M. Thormann , M. Hinnerichs , et al., “Impact of Body Composition in Advanced Hepatocellular Carcinoma: A Subanalysis of the SORAMIC Trial,” Hepatol Commun. 7, no. 6 (2023): e0165. 37219875 10.1097/HC9.0000000000000165 PMC10208699

[11] A. Surov , M. Hinnerichs , I. Shahzadi , et al., “Parameters of Body Composition Predict Clinical Course in Acute Colonic Diverticulitis,” Journal of Cachexia, Sarcopenia and Muscle 16, no. 3 (2025): e13864. 40524380 10.1002/jcsm.13864 PMC12170454

[12] B. A. Derstine , S. A. Holcombe , B. E. Ross , N. C. Wang , G. L. Su , and S. C. Wang , “Skeletal Muscle Cutoff Values for Sarcopenia Diagnosis Using T10 to L5 Measurements in a Healthy US Population,” Scientific Reports 8, no. 1 (2018): 11369. 30054580 10.1038/s41598-018-29825-5 PMC6063941

[13] A. Surov , J. Borggrefe , A. K. Höhn , and H. J. Meyer , “Associations Between ADC Histogram Analysis Values and Tumor‐Micro Milieu in Uterine Cervical Cancer,” Cancer Imaging 24, no. 1 (2024): 170, 10.1186/s40644-024-00814-4. 39707580 PMC11662562

[14] J. Kasurinen , J. Hagström , T. Kaprio , I. Beilmann‐Lehtonen , C. Haglund , and C. Böckelman , “Tumor‐Associated CD3‐ and CD8‐Positive Immune Cells in Colorectal Cancer: The Additional Prognostic Value of CD8+‐to‐CD3+ Ratio Remains Debatable,” Tumour Biology 44, no. 1 (2022): 37–52, 10.3233/TUB-211571. 35404299

[15] S. Timmers , P. Schrauwen , and J. de Vogal , “Muscular Diacylglycerol Metabolism and Insulin Resistance,” Physiology & Behavior 94 (2008): 242–251. 18207474 10.1016/j.physbeh.2007.12.002

[16] M. Jung , H. Rieder , M. Reisert , et al., “Association Between Myosteatosis and Impaired Glucose Metabolism: A Deep Learning Whole‐Body Magnetic Resonance Imaging Population Phenotyping Approach,” Journal of Cachexia, Sarcopenia and Muscle 15, no. 5 (2024 Oct): 1750–1760. 39009381 10.1002/jcsm.13527 PMC11446675

[17] M. J. Kim , Y. K. Cho , H. N. Jung , et al., “Association Between Insulin Resistance and Myosteatosis Measured by Abdominal Computed Tomography,” Journal of Clinical Endocrinology and Metabolism 108, no. 12 (2023): 3100–3110. 37401630 10.1210/clinem/dgad382

[18] M. M. Ibrahim , “Subcutaneous and Visceral Adipose Tissue: Structural and Functional Differences,” Obesity Reviews 11, no. 1 (2010): 11–18, 10.1111/j.1467-789X.2009.00623.x. 19656312

[19] J. P. Després and I. Lemieux , “Abdominal Obesity and Metabolic Syndrome,” Nature 444, no. 7121 (2006): 881–887, 10.1038/nature05488. 17167477

[20] Y. Okugawa , Y. Toiyama , A. Yamamoto , et al., “Close Relationship Between Immunological/Inflammatory Markers and Myopenia and Myosteatosis in Patients With Colorectal Cancer: A Propensity Score Matching Analysis,” JPEN Journal of Parenteral and Enteral Nutrition 43, no. 4 (2019): 508–515. 30334265 10.1002/jpen.1459

[21] Z. Zhao , H. Ding , Z. B. Lin , et al., “Relationship Between Tertiary Lymphoid Structure and the Prognosis and Clinicopathologic Characteristics in Solid Tumors,” International Journal of Medical Sciences 18, no. 11 (2021): 2327–2338. 33967609 10.7150/ijms.56347 PMC8100653

[22] M. J. Gooden , G. H. de Bock , N. Leffers , T. Daemen , and H. W. Nijman , “The Prognostic Influence of Tumour‐Infiltrating Lymphocytes in Cancer: A Systematic Review With Meta‐Analysis,” British Journal of Cancer 105, no. 1 (2011): 93–103. 21629244 10.1038/bjc.2011.189 PMC3137407

[23] B. S. Nedergaard , M. Ladekarl , H. F. Thomsen , J. R. Nyengaard , and K. Nielsen , “Low Density of CD3+, CD4+ and CD8+ Cells is Associated With Increased Risk of Relapse in Squamous Cell Cervical Cancer,” British Journal of Cancer 97, no. 8 (2007): 1135–1138. 17940503 10.1038/sj.bjc.6604001 PMC2360435

[24] A. Ohno , T. Iwata , Y. Katoh , et al., “Tumor‐Infiltrating Lymphocytes Predict Survival Outcomes in Patients With Cervical Cancer Treated With Concurrent Chemoradiotherapy,” Gynecologic Oncology 159, no. 2 (2020): 329–334. 32829964 10.1016/j.ygyno.2020.07.106

[25] J. Wang , Z. Li , A. Gao , Q. Wen , and Y. Sun , “The Prognostic Landscape of Tumor‐Infiltrating Immune Cells in Cervical Cancer,” Biomedicine & Pharmacotherapy 120 (2019 Dec): 109444. 31562978 10.1016/j.biopha.2019.109444

[26] Y. Miyasaka , Y. Yoshimoto , K. Ando , et al., “CD8‐Positive Tumor‐Infiltrating Lymphocytes and Prognosis in Radiotherapy for Uterine Cervical Squamous Cell Carcinoma,” Anticancer Research 43, no. 5 (2023): 2077–2084. 37097697 10.21873/anticanres.16368

[27] S. Y. Park , B. O. Hwang , and N. Y. Song , “The Role of Myokines in Cancer: Crosstalk Between Skeletal Muscle and Tumor,” BMB Reports 56, no. 7 (2023): 365–373. 37291054 10.5483/BMBRep.2023-0064 PMC10390289

[28] C. Conlon , E. Lugli , H. C. Welles , et al., “Redistribution, Hyperproliferation, Activation of Natural Killer Cells and CD8 T Cells, and Cytokine Production During First‐in‐Human Clinical Trial of Recombinant Human Interleukin‐15 in Patients With Cancer,” Journal of Clinical Oncology 33, no. 1 (2015): 74–82. 25403209 10.1200/JCO.2014.57.3329 PMC4268254

[29] C. Ravensbergen , R. van Kooten , S. Crobach , et al., “Association Between Muscle Mass, Visceral Adiposity, and Histologic Tumor Stromal Features in Colon Cancer,” Clinical Nutrition ESPEN 65 (2025): 282–287. 39662586 10.1016/j.clnesp.2024.12.012

[30] Y. Kitano , Y. I. Yamashita , Y. Saito , et al., “Sarcopenia Affects Systemic and Local Immune System and Impacts Postoperative Outcome in Patients With Extrahepatic Cholangiocarcinoma,” World Journal of Surgery 43, no. 9 (2019): 2271–2280. 31041559 10.1007/s00268-019-05013-y

[31] S. Doi , S. Yasuda , Y. Matsuo , et al., “Clinical Impact of Sarcopenia in Early‐Stage Intrahepatic Recurrent Hepatocellular Carcinoma: An Association With Impaired Host Immunity,” Langenbeck's Archives of Surgery 408, no. 1 (2023): 433. 10.1007/s00423-023-03170-2 37950033

[32] S. Masuda , K. Yamakawa , A. Masuda , et al., “Association of Sarcopenia With a Poor Prognosis and Decreased Tumor‐Infiltrating CD8‐Positive T Cells in Pancreatic Ductal Adenocarcinoma: A Retrospective Analysis,” Annals of Surgical Oncology 30, no. 9 (2023): 5776–5787. 37191859 10.1245/s10434-023-13569-2 PMC10409680

[33] N. Daitoku , Y. Miyamoto , Y. Hiyoshi , et al., “Preoperative Skeletal Muscle Status Is Associated With Tumor‐Infiltrating Lymphocytes and Prognosis in Patients With Colorectal Cancer,” Annals of Gastroenterological Surgery 6, no. 5 (2022): 658–666. 36091309 10.1002/ags3.12570 PMC9444852

[34] G. Hu and S. Wang , “Tumor‐Infiltrating CD45RO(+) Memory T Lymphocytes Predict Favorable Clinical Outcome in Solid Tumors,” Scientific Reports 7, no. 1 (2017): 10376. 28871164 10.1038/s41598-017-11122-2 PMC5583330

[35] Y. Kim , Y. Shin , and G. H. Kang , “Prognostic Significance of CD103+ Immune Cells in Solid Tumor: A Systemic Review and Meta‐Analysis,” Scientific Reports 9, no. 1 (2019): 3808. 30846807 10.1038/s41598-019-40527-4 PMC6405906

[36] Q. Jia , Y. Yang , and Y. Wan , “Tumor‐Infiltrating Memory T‐Lymphocytes for Prognostic Prediction in Cancer Patients: A Meta‐Analysis,” International Journal of Clinical and Experimental Medicine 8, no. 2 (2015): 1803–1813. 25932108 PMC4402755

[37] G. Hu and S. Wang , “Prognostic Role of Tumor‐Infiltrating CD57‐Positive Lymphocytes in Solid Tumors: A Meta‐Analysis,” Oncotarget 9, no. 8 (2017): 8111–8119. 29487719 10.18632/oncotarget.23621 PMC5814286

[38] H. Liu , Z. Li , X. Han , et al., “The Prognostic Impact of Tumor‐Infiltrating B Lymphocytes in Patients With Solid Malignancies: A Systematic Review and Meta‐Analysis,” Critical Reviews in Oncology/Hematology 181 (2023): 103893. 36481308 10.1016/j.critrevonc.2022.103893

[39] S. Zhang , W. Liu , B. Hu , et al., “Prognostic Significance of Tumor‐Infiltrating Natural Killer Cells in Solid Tumors: A Systematic Review and Meta‐Analysis,” Frontiers in Immunology 11 (2020): 1242. 32714321 10.3389/fimmu.2020.01242 PMC7343909

[40] L. J. Halliday , P. R. Boshier , E. Doganay , V. Wynter‐Blyth , J. P. Buckley , and K. Moorthy , “The Effects of Prehabilitation on Body Composition in Patients Undergoing Multimodal Therapy for Esophageal Cancer,” Diseases of the Esophagus 36, no. 2 (2023): doac046. 35795994 10.1093/dote/doac046 PMC9885737

